# 

**Published:** 1893-12-09

**Authors:** 


**Notice to Advertisers and Subscribers.**

All Advertisements, Orders for Copies of The Hospital, and Business Communications should be addressed to The Publisher, *not to the Editor*, The Hospital, 428, Strand, W.C.

The Cost of Subscription, post free, is as follows:—Per week, 2½d.; per quarter, 2s. 6d.; per half-year, 5s.; per annum, 8s. 6d Foreign:—Per Annum, 10s. 8d.; payable, in all cases, in advance.

**OUR NEW OFFICES.**

428, Strand, W.C., will henceforth be the Office of this Journal.

**Notices of Births, Deaths, and Marriages** are inserted in this column at a charge of 2s. 6d. for an announcement not exceeding four lines.

**NOTICE TO CORRESPONDENTS.**

All MS., Letters, Books for Review, and other matters intended for the Editor should be addressed The Editor, The Lodge, Porchester Square, London, W.

The Editor cannot undertake to return rejected MS., even when accompanied by stamped directed envelope.

**"Burdett's Hospital Annual," 1893.**

Fourth year of publication. 700 pp. Boards, gilt lettering. A most complete guide to British, Colonial, Indian, and American Hospitals, Dispensaries, Nursing and Convalescent Institutions, and Asylums. Price 5s. Published by The Scientific Press (Limited), 428, Strand, W.C.

**NOTICE.—The Index for Vol. XIV. (ending Sept. 30th, 1893) is on sale at the Office of this Journal, price 2½d., post free.**

### SCRAPS AND GLEANINGS.

The directors of Leith Hospital have before them at present plans for the reconstruction of that part of the institution which formerly was devoted to the treatment of the cases of infectious disease occurring in the burgh. By this change six fever wards and a probationary ward are at the disposal of the directors.

As was fully anticipated, the annual charge of the Aberdeen Royal Infirmary, under the Jubilee scheme of extension and improvement, by much the heavier part of which has now been accomplished, has increased by something like £1,500 a year.

### PUBLISHERS' ANNOUNCEMENTS.

Pocket size, 2s. post free, with numerous Illustrations, reduced from the larger work by the same Author.

**A PRIMER OF THE ART OF MASSAGE (FOR LEARNERS).**

By THOMAS STRETCH DOWSE, M.D., F.R.C.P.Edin.

"An excellent little book, giving the more important facts about massage. Will be useful."—*Dub. Med. Jour.*

Bristol: J. Wright and Co.

**NOW READY.**

2/6 Post Free. *THIRD EDITION, enlarged and partly re-written, cloth gilt, crown 8vo., about 200 pp.* 2/6 Post Free.

**HOSPITAL SISTERS AND THEIR DUTIES.**

BY

**EVA C. E. LÜCKES**

(*Matron of the LONDON HOSPITAL*).

London: The Scientific Press (Limited), 428, Strand, W.C.

200 pp., Crown 8vo, profusely illustrated with original drawings. List of Instruments required, and a Glossary of Terms, cloth gilt, 3s. 6d.

**OPHTHALMIC NURSING,**

By SYDNEY STEPHENSON, M.B., F.R.C.S.E., Surgeon to the Ophthalmic School, Hanwell, W., etc., etc.

The first work on this important branch of Nursing.

Expounds clearly the principles of Nursing Eye Diseases.

Most of the illustrations were specially prepared for this work.

Ophthalmic Institutions will find it invaluable as a text-book for the training of their Nurses.

*Nursing Notes*, December, 1893, says:—"A book on Ophthalmic Nursing has been a real need, which is now well supplied." . . . "Those contemplating going in for Ophthalmic Nursing should study this useful work." . . . "One of the most useful books for Nurses we have seen for a long time."

London: The Scientific Press (Limited), 428, Strand, W.C.

**BOOKS FOR NURSES.**

**SURGICAL WARD WORK AND NURSING.** By ALEX. MILES, M.D., &c., &c. 200 pp., demy 8vo., cloth gilt, copiously illustrated, price 3s. 6d.

**ART OF MASSAGE.** By A. CREIGHTON HALE. Demy 8vo., nearly 200 pp., cloth gilt, profusely illustrated with over 70 special cuts, price 6s. (post free).

As a text-book this volume will be found invaluable. It will be found to contain a complete exposition of the Art of Massage, together with many useful hints and much valuable information which every Masseuse should read.

**HOW TO BECOME A NURSE; AND HOW TO SUCCEED.** Compiled by HONNOR MORTEN. Demy 8vo., 200 pp., Fourth Thousand, profusely illustrated with Copyright Portraits and Drawings, price 2s. 6d. (post free).

A complete guide to the nursing profession for those who wish to become Nurses, and a useful book of reference for Nurses who have completed their training and seek employment.

**ART OF FEEDING THE INVALID.** By a MEDICAL PRACTITIONER and a LADY PROFESSOR of COOKERY. Demy 8vo., cloth gilt, pp. 260. Price 3s. 6d.

**"THE HOSPITAL" NURSE'S CASE BOOK.** For use in hospital as well as private nursing. Demy 16mo. (for the apron pocket), 72 pp., strong boards, Second Edition, price 6d., or 4s. 6d. per dozen (post free).

A book of tables specially prepared for use by nurses in the ward and in the sick-room.

**THE NURSES' DICTIONARY OF MEDICAL TERMS AND NURSING TREATMENT.** Compiled for the use of nurses by HONNOR MORTEN. Second and Revised Edition, demy 16mo. (suitable for the apron pocket), handsomely bound in terra-cotta cloth boards, 140 pp., price 2s., in leather, price 2s. 6d.

Publishers: The Scientific Press (Limited), 428, Strand, London, W.C.

### BOOKS RECEIVED.

LONGMANS, GREEN, AND CO.

"The True Story Book." Edited by Andrew Lang.
"The Outdoor World; or the Young Collector's Handbook." By W. Furneaux, F.R.G.S.

CHATTO AND WINDUS.

"St. Katherine's Tower." By Walter Besant.
"Heather and Snow," A Novel. By George MacDonald.
"To Let." By B. M. Croker.
"Rujub, the Juggler." By G. A. Henty.
"The Master of St. Benedict's. By Alan St. Aubyn.

CASSELL AND CO.

"Little Folks," for 1893.
"Bo Peep," for 1893.
"The Quiver," for 1893.

BLACKIE AND SON.

"Nicola; or the Career of a Girl Musician." By M. Corbet-Seymour.
"Westward with Columbus." By Gordon Stables, M.D.
"A True Cornish Maid." By G. Norway.

DIGBY, LONG, AND CO.

"Our Ghosts." By Edmund Leigh.

T. FISHER UNWIN.

"Wild Nature Won by Kindness." By Mrs. Brightwen.

JOHN BALE AND SONS.

"Rheumatism; some Investigations Respecting its Cause, Prevention and Cure." By Percy Wilde, M.D.

### Medical Vacancies and Appointments.

### WESTMINSTER HOSPITAL STUDENTS' DINNER.

A Correction.—Mr. De Rienzi writes: In the account of the above dinner which is contained in your issue of November 25th, I am reported as follows: "Allusion was made to the nursing of the hospital, and it was suggested that a more frequent change of probationers in the wards might be beneficial." I beg to state that such an idea was the very reverse of what it was my intention to convey. What I said was, that "a less frequent change would be beneficial."

### APPOINTMENTS.

H. N. Crawley Atkinson, M.R.C.S.Eng., L.R.C.P.Lond., appointed Junior Resident Medical Officer (House Physician) to the Royal Free Hospital, Gray's Inn Road. J. Dunnell Rawlings, M.R.C.S.Eng., L.R.C.P.Lond., appointed Junior Resident Medical Officer (House Physician) to the Royal Free Hospital, Gray's Inn Road.

### VACANCIES.

The following vacancies are announced this week, the amount of salary in each case being given after the name of the institution:

#### Resident Medical Officers.

Bradford Temporary Small-Pox Hospital. Resident Medical Officer. Salary at the rate of £150 per annum, with board, lodging, and washing. Applications to the Medical Officer of Health, Town Hall, Bradford.

City of London Hospital for Diseases of the Chest, Victoria Park, E. House Physicians. Board and residence, and allowance for washing provided. Appointment for six months. Applications to the Secretary at the Office, 24, Finsbury Circus, E.C., by December 14th.

City of London Lying-In Hospital, City Road, E.C. District Surgeons. Applications to R. A. Owthwaite, Secretary, by December 19th.

Dudley Dispensary. Resident Medical Officer; doubly qualified. Salary £130, with increase at the end of twelve months if the services are satisfactory. Good house, coal, gas, and water found, and all rates and taxes paid. Applications to H. C. Brettell, Honorary Secretary, by December 11th.

Kimberley Hospital, Kimberley. Senior House Surgeon. Salary £650 per annum, with unfurnished quarters for single men. Applications to H. A. De Beer, Secretary, by December 25th.

North-Eastern Hospital for Children, Hackney Road, N.E. House Surgeon; doubly qualified. Appointment for six months, and at the expiration of that time will be required, if eligible, to serve as Senior House Surgeon for a further period of six months. Salary for the junior post at the rate of £60 per annum, and for the senior post at the rate of £80 per annum. Applications to the Secretary, 27, Clement's Lane, E.C.

Royal Free Hospital, Gray's Inn Road, W.C. Senior Resident Medical Officer; doubly qualified. Salary £100 per annum, with board, residence, and washing. Appointment for twelve months, but eligible for re-election. Applications to the Secretary by December 11th.

Royal Free Hospital, Gray's Inn Road, W.C. Junior Resident Medical Officer (House Surgeon); doubly qualified. Board, residence, and washing. Canvassing members of the Board not permitted. Applications to Conrad W. Thies, Secretary, by December 16th.

#### Non-Resident Medical Officer.

Dental Hospital of London, Leicester Square, W.C. Demonstrator. Honorarium £50 per annum. Applications to Mr. Morton Smale, Dean, by December 18th.

### PASS LISTS.

#### University of London.

The following gentlemen have passed the M.B. Examination, held in October, 1893.

First Division.—H. W. Armstead, University College and St. Bartholomew's Hospital; T. Carwardine, Middlesex Hospital and University College; J. G. Clegg, Owens College and Manchester Royal Infirmary; H. O. Davies, St. Bartholomew's Hospital; H. G. Felkin, London Hospital; S. F. Gibbs, St. Bartholomew's Hospital; J. A. Howard, Guy's Hospital; C. S. Jaffé, St. Thomas's Hospital; W. J. Johnson, Guy's Hospital; Lillie Mabel A. Jones, London School of Medicine and Royal Free Hospital; B. H. F. Leumann, St. Bartholomew's Hospital; E. G. G. Little, St. George's Hospital; J. Morrison, St. Bartholomew's Hospital; J. L. Morton, St. Mary's Hospital; E. A. Nathan, St. Mary's Hospital; A. P. Nuttall, University College; J. A. Pickels, Owens College and Manchester Royal Infirmary; W. J. Potts, Owens College and Manchester Royal Infirmary; F. E. Rock, Middlesex Hospital; K. Rogers, St. Bartholomew's Hospital; W. N. Soden, St. Bartholomew's Hospital; J. C. Symes, St. Mary's Hospital; H. C. Thomson, Middlesex Hospital; W. L. Wainwright, St. Thomas's Hospital; W. B. Warrington, Owens College and Manchester Royal Infirmary; C. J. Woollett, Charing Cross Hospital.

### The Hospital Advertiser.

### MEDICAL APPOINTMENTS.

CITY OF LONDON LYING-IN HOSPITAL, CITY ROAD, E.C.

DISTRICT SURGEONS.

VACANCIES have occurred in Upper Holloway and N. Division of Bethnal Green, including Hackney.

Applications for the posts, stating qualifications, &c., must be sent to the undersigned not later than TUESDAY, the 19th December.

R. A. OWTHWAITE, Secretary.

SECOND DIVISION.—M. Ashley, University College; L. W. Bathurst, St. Bartholomew's Hospital; W. Branson, Sheffield and University College; S. M. Brown, Owens College and Manchester Royal Infirmary; J. LeMare Bunsh, B.Sc., University College; D. A. Channing-Pearce, Guy's Hospital; D. W. Collings, St. Bartholomew's Hospital; R. H. Crowley, St. Bartholomew's Hospital; W. E. De Korté, Guy's Hospital; J. Dickinson, Westminster Hospital; P. S. Eves, University College; J. H. Griffiths, St. Bartholomew's Hospital; J. L. Iredale, Yorkshire College; T. T. Jeans, Owens College and Manchester Royal Infirmary; G. P. Jerome, Queen's College, Birmingham; A. W. Kirkpatrick-Picard, University College; C. A. Lane, St. Mary's Hospital; F. W. Lewis, St. Mary's Hospital; G. F. Murrell, St. Bartholomew's Hospital; E. A. Perram, St. Bartholomew's Hospital; Ann Frances Piercy, London School of Medicine and Royal Free Hospital; F. P. Piper, St, Mary's Hospital; T. M. J. Powell, St. Bartholomew's Hospital; E. J. Smyth, B.Sc., University College; T. B. Stedman, University College; J. P. Tildesley, Queen's Hospital and College, Birmingham; F. T. Travers, University College; B. P. Viret, St. Bartholomew's Hospital; S. R. Wells, B.Sc., St. George's Hospital; J. Ryland Whitaker, B.A., University College; A. E. Wilson, St. Mary's Hospital; S. F. Wright, St. Thomas's Hospital.

### THE ROYAL COLLEGE OF PHYSICIANS, EDINBURGH. TRIPLE QUALIFICATION.

### Papers Recently Set.

FINAL EXAMINATION.

QUESTIONS IN MEDICINE. (Four Questions, of which Three only are to be answered.)

1. Describe the symptoms and physical signs which you would expect to be present in a case of large Aneurism of the Abdominal Aorta.
2. What is the pathology of Myxœdema? Describe the symptoms which you would expect to be present in a typical case of Myxœdema.
3. What are the causes of Waxy (Amyloid) Degeneration? What organs are most frequently affected by Amyloid Degeneration? What symptoms and physical signs would warrant a diagnosis of Amyloid Degeneration during life?
4. Describe the symptoms which would result from complete destruction of the right third nerve at the base of the brain.

QUESTIONS IN THERAPEUTICS. (All of which are to be answered, including the Prescription.)

1. Describe the treatment which you would recommend in a case of Tubercular Laryngitis.
2. How would you treat a case of Megrim (Sick-Headache)?
3. Describe the treatment which you would advise in a case of Multiple Osteoid Arthritis.

PRESCRIPTION. (To be written in unabbreviated Latin, and the directions in English.)

Prescribe a pill containing aloin, nux vomica, ipecacuahna, and belladonna, for a case of Chronic Constipation.

QUESTIONS IN SURGERY. (Four Questions, of which Three only are to be answered.)

1. What are the anatomical conditions which render Concussion of the Spinal Cord a rare injury?
2. In what circumstances do Bed Sores occur? How are they prevented, and how treated?
3. What are the causes, symptoms, and treatment of Atony of the Bladder?
4. In what conditions is Bone-grafting useful, and how should it be performed?

SURGICAL ANATOMY. (Both of which are to be answered.)

1. What are the relations of the Femoral Artery in Scarpa's Triangle?
2. Describe the ligaments of the knee-joint.

QUESTIONS IN MIDWIFERY AND GYNÆCOLOGY. (Only Three Questions to be answered, of which the Fourth must be one.)

1. Describe the principal deformities of the brim of the Pelvis due to Rachitis.

2. Describe the means available, at an early stage of Labour, for diagnosing a Transverse Position of the Fœtus.

3. Describe the two kinds of Hæmorrhage which may occur during Pregnancy, and state their more obvious causes.

4. How is Prolapsed Cystic Ovary diagnosed during Labour, and what is the treatment?

QUESTIONS IN MEDICAL JURISPRUDENCE AND HYGIENE. (Only Three Questions to be answered, including Nos. 3 and 4.)

1. Contrast the external and internal *post-mortem* appearances found on a Body when death has been caused by Strangulation, with those of death produced by Hæmorrhage.
2. Describe the nature of the Lesions which may be produced on a living person (1) by fire; and (2) by corrosives. What are the points of difference between them, and between similar lesions produced after death?
3. Name the Medicinal and other agents which might be used criminally as abortifacients? Describe how they act, and how you would detect the medicinal agents in the body after death.
4. What Diseases may arise from overcrowding? What cubic space per individual does the law demand in schools and lodging-houses? What tests would you employ to discover the quality of the atmosphere of an overcrowded room?

